# Epidemiological Characteristics of Clinical *Aspergillus* Section *Nigri* and Mutations in *cyp51A* Gene

**DOI:** 10.3390/jof12090683

**Published:** 2026-09-11

**Authors:** Yuyi Zhang, Yao Zhang, Suzhen Wang, Chunmei Zhou, Jue Pan, Wei Guo, Bijie Hu

**Affiliations:** 1 Department of Laboratory Medicine, Zhongshan Hospital, Fudan University, Shanghai 200032, China; zhang.yuyi@zs-hospital.sh.cn (Y.Z.);; 2 Department of Infectious Disease, Zhongshan Hospital, Fudan University, Shanghai 200032, China; zhang.yao@zs-hospital.sh.cn (Y.Z.);

**Keywords:** *Aspergillus* section *Nigri*, azole resistance, *cyp51A* gene, Y119F mutation

## Abstract

*Aspergillus* section *Nigri* is distributed ubiquitously in nature and is reported to be the third most clinically significant species of the *Aspergillus* genus. In this study, we surveyed *Aspergillus* section *Nigri* isolates from Zhongshan Hospital, Fudan University, Shanghai, China, from 2019 to 2023. We determined the species distribution of section *Nigri* isolates by partial *β-tubulin* gene sequencing. We characterized the antifungal susceptibility profiles of these isolates to medical azoles (itraconazole, voriconazole, posaconazole, and isavuconazole) using the Sensititre^TM^ custom-designed broth microdilution system. The *cyp51A* sequences were compared between azole-resistant and azole-susceptible strains in order to determine the potential antifungal resistance mechanism. A total of 54 *Aspergillus* section *Nigri* isolates were obtained. *A. tubingensis* (50.0%) and *A. niger* (46.3%) were the most prevalent species. Two isolates of *A. welwitschiae* were identified. Resistance to Itraconazole, voriconazole, and isavuconazole was found in 26%, 33.3%, and 33.3% of *A. tubingensis* isolates, respectively. All *A. niger* and *A. welwitschiae* isolates were sensitive to these azoles. Three amino acid mutations were found in azole-resistant *A. tubingensis* isolates, namely Y119F, Y119H, and T321A. Results from this study provide us with an understanding of species distribution and antifungal susceptibility profiles of clinical *Aspergillus* section *Nigri* isolates in Eastern China. It highlights the importance of accurate species identification and antifungal susceptibility testing. Our results also suggest that the novel Y119F substitution is potentially a key player in azole resistance.

## 1. Introduction

*Aspergillus* species are distributed ubiquitously in nature, widely found in air and decomposing organic matter. They stand out as an important cause of opportunistic fungal diseases. Due to the increased number of immunocompromised individuals, *Aspergillus* species have emerged as a major global health concern. An extensive literature review including data from over 120 countries (2010 to 2023) estimated that more than 2 million people develop invasive aspergillosis (IA) each year, with a crude mortality of 1.8 million deaths [1]. *Aspergillus fumigatus* remains the most frequently isolated species from aspergillosis patients. A systematic literature search from 2016 to 2021 included 49 studies [2]. The twelve-week mortality rates in IA cases ranged from 18% to 54.5%, depending on patient population (e.g., hematology patients had significantly higher mortality rates) and azole resistance. Hence, *A. fumigatus* has been listed by the World Health Organization as a critical priority fungal pathogen.

While *A. fumigatus* is the primary focus of clinical attention and research, recent years have seen an increase in infections due to non-*fumigatus Aspergillus* species [3]. Species belonging to *Aspergillus* section *Nigri* are often reported to be the second most common non-*fumigatus Aspergillus* species isolated from clinical specimens [3,4]. They cause fungal infections in humans (approximately 5–22.3% of aspergillosis, depending on geographical regions), which include a broad spectrum of illnesses from otomycosis to life-threatening IA [5]. The prevalence has been markedly increased. A study in Belgium observed that invasive infections caused by section *Nigri* isolates increased fourfold from 2005 to 2011 [6]. *Aspergillus* section *Nigri* (often known as “black aspergilli”) comprises 27 species, including *A*. *niger* and its related cryptic species [7]. The taxonomy of *Aspergillus* section *Nigri* is morphologically indistinguishable and is considered a homogeneous species [8]. They are often reported as *A. niger* or *A. niger* complex in the clinical laboratory based on traditional culture-based methods. In recent years, non-culture-based techniques (proteomic and molecular methods) have refined the taxonomy of *Aspergillus* section *Nigri* [7]. However, little is known about the species distribution due to the lack of accurate species identification, especially in low-resource regions. Misidentification and understudy of these species may compromise proper treatment and prognosis.

Mold-active triazoles (itraconazole, posaconazole, voriconazole, and isavuconazole) are first-line therapy for IA and chronic pulmonary aspergillosis. They act by inhibiting the biosynthesis of lanosterol 14-α-demethylase (CYP51A), which in turn interrupts cell growth [3]. Azole resistance in *Aspergillus* species has emerged globally and has become a formidable challenge to effective aspergillosis treatment. In contrast, antimycotics from other classes, including echinocandins and amphotericin B, serve as alternative or combination therapeutic options for aspergillosis [3]. They generally maintain potent activity against most species, with rare reports of resistance except for intrinsic resistance in a small subset of species [8]. For this reason, most antifungal surveillance studies have focused primarily on azole resistance. Azole resistance in *A. fumigatus* was intensively studied due to its global prevalence. The prevalence of azole-resistant *A. fumigatus* was reported in clinical and environmental studies, ranging from 0.3% to 47.1% [9]. Cyp51 protein alteration and *cyp51* gene overexpression constitute the most important mechanisms described [9]. On the other hand, non-*fumigatus Aspergillus* species have demonstrated variation in in vitro antifungal susceptibility [10]. Numerous studies have reported that *A*. *tubingensis* in the *Nigri* section showed higher minimum inhibitory concentration (MIC) to itraconazole and/or voriconazole [11,12]. Furthermore, this less susceptible species has been increasingly recognized as an important clinical pathogen. Recent epidemiological studies observed that *A. tubingensis* was equally or even more prevalent than *A. niger* in clinical settings [11,12]. However, the underlying genetic basis of variation in azole resistance in the section *Nigri* remains unresolved [13,14].

In Eastern China, *Aspergillus* section *Nigri* is the third most frequently isolated *Aspergillus* spp. from clinical specimens, following *A. fumigatus* and *A*. *flavus* [4]. Yet, detailed epidemiology and antifungal susceptibility patterns of these strains have not been reported due to their rare occurrence in clinical settings. In this study, we surveyed *Aspergillus* section *Nigri* isolates recovered from Zhongshan Hospital, Fudan University, Shanghai, China, from 2019 to 2023. We aimed to investigate the detailed epidemiology and potential underlying azole resistance mechanisms. Accurate identification at the species level and evaluation of their susceptibility profile are critical to determine optimal treatment, which in turn improves patient outcomes.

## 2. Materials and Methods

### 2.1. Clinical Data Collection

All *Aspergillus* section *Nigri* isolates obtained from respiratory samples for routine diagnostic culture examination were collected at one of the largest tertiary teaching hospitals in China, Zhongshan Hospital, Fudan University, from 2019 to 2023. Probable invasive aspergillosis (IA) and chronic pulmonary aspergillosis (CPA) were classified according to the European Organization for Research and Treatment of Cancer and the Mycoses Study Group (EORTC/MSG) definitions proposed in 2020, the European Society for Clinical Microbiology and Infectious Diseases, the European Confederation of Medical Mycology and the European Respiratory Society Joint Clinical Guidelines (ESCMID-ECMM-ERS) proposed in 2017 [15,16]. COVID-19-associated pulmonary aspergillosis was defined according to the European Confederation of Medical Mycology and the International Society for Human and Animal Mycology (ECMM/ISHAM) consensus criteria proposed in 2020 [17].

### 2.2. Isolate Identification

All samples were processed according to routine clinical mycological procedures. Recovered section Nigri isolates were identified using Matrix-Assisted Laser Desorption Ionization-Time of Flight Mass Spectrometry (MALDI-ToF-MS) (bioMérieux, Marcy-l’Étoile, France) according to the manufacturer’s protocol. Briefly, well-isolated colonies were inoculated into a 1.5 mL Eppendorf tube containing 900 µL of 70% ethanol and vortexed. The ethanol was fully removed from the fungal pellet after 3 min of centrifugation at 13,000× *g*. A total of 40 µL of absolute acetonitrile was added to the pellet and vortexed. After that, 70% formic acid was added and vortexed. One µL of supernatant was dispensed onto the MS target (bioMérieux, Marcy-l’Étoile, France) after 3 min of centrifugation at 13,000× *g*. It was then covered with 1 µL of MS matrix (bioMérieux, France). Identification to the species level was ensured by partial *β-tubulin* gene sequencing [4]. DNA was extracted from the mycelia using TIANamp Yeast DNA Kit (TIANGEN, Beijing, China). Polymerase chain reaction amplification and sequencing were performed with the primer pair of Bt2A_F (5′-AATTGGTGCCGCTTTCTGG-3′) and Bt2A_R (5′-AGCGTCCATGGTACCGG-3′). The DNA sequences of these isolates were analyzed using CLC Main Workbench (QIAGEN Aarhus, Aarhus, Denmark) and compared to sequences deposited in the NCBI GenBank database using the Basic Local Alignment Search Tool (BLAST+ v2.16.0; https://blast.ncbi.nlm.nih.gov/Blast.cgi (accessed on 10 September 2025).

### 2.3. In Vitro Susceptibility Testing

Antifungal drug susceptibility was evaluated on all *Aspergillus* section *Nigri* isolates against four medical azoles, namely itraconazole (IZ), voriconazole (VOR), posaconazole (POS), and isavuconazole (ISA), using the Sensititre^TM^ custom-designed broth microdilution system (Thermo Fisher Scientific, Waltham, MA, USA) according to the manufacturer’s protocol. Briefly, well-isolated colonies from sporulated cultures of section *Nigri* isolates were inoculated into sterile saline water. Conidial suspension was adjusted spectrophotometrically to a turbidity of 0.5 McFarland (0.6–5.0 × 106 CFU/mL). It was then diluted with YeastOne^TM^ inoculum broth (Thermo Fisher Scientific, USA) and mixed thoroughly. The mixture was further inoculated into each well of the Sensititre^TM^ custom-designed microdilution plate (Thermo Fisher Scientific, USA). The reference *A. fumigatus* isolate ATCC 204305, *Candida parapsilosis* isolate ATCC 22019, and *Candida krusei* isolate ATCC 6258 were used as quality control strains. MIC values were determined after 48 h of incubation at 35 °C. For *A. niger* isolates, the azole wild-type (susceptible) and non-wild-type (resistant) were classified according to the epidemiological cutoff values (ECVs) proposed by the Clinical and Laboratory Standards Institute (CLSI) guideline M59 [18]. *A. niger* isolates were regarded as resistant if they had itraconazole, voriconazole, posaconazole, and isavuconazole MIC values above 4 μg/mL, 2 μg/mL, 2 μg/mL and 4 μg/mL, respectively. In the absence of clinical breakpoints (CBPs) and ECVs for these cryptic species, proposed ECVs for *A. niger* were applied to this data set for azoles.

### 2.4. Detection of cyp51A Mutations

In order to determine the potential genetic mechanisms underlying azole resistance in section *Nigri* isolates, the entire coding region of the *cyp51A* gene was analyzed as described previously [4]. Polymerase chain reaction amplification and sequencing were performed with the primer pair of Cyp51A_F (5′-TTCGCAACGAGAAAGAACCAC-3′) and Cyp51A_R (5′-CTCCCAGCCCACGATAGCAG-3′). The sequence of *cyp51A* of the azole-susceptible strain P53-27176 was used as the reference sequence. Sequences of *cyp51A* from azole-resistant strains were aligned and compared with the reference sequence using CLC Main Workbench (QIAGEN Aarhus, Aarhus, Denmark).

### 2.5. Ethics Statement

This study was approved by the Ethics Committee of Zhongshan Hospital, Fudan University (approval number: B2022-376R).

## 3. Results

### 3.1. Clinical Origin and Species Composition

A total of 54 *Aspergillus* section *Nigri* isolates from 53 patients were obtained from 2019 to 2023. Two *A. tubingensis* strains were isolated from one patient as they exhibited distinct azole susceptibility profiles. All of these section *Nigri* isolates were recovered from respiratory specimens, including sputum (n = 52) and bronchoalveolar lavage fluid (n = 2). Most of the patients enrolled in our study were from Eastern China: 52.8% from Shanghai, 18.9% from Jiangsu province, and 3.8% from Zhejiang province. Twelve patients were diagnosed with chronic pulmonary aspergillosis. Ten patients were diagnosed with probable invasive aspergillosis. Nine and one patient were diagnosed with COVID-19-associated pulmonary aspergillosis and influenza-associated pulmonary aspergillosis, respectively. The remaining patients (n = 21) were found to be colonized with section *Nigri* isolates.

All isolates were identified as *A. niger* complex using MALDI-ToF-MS (bioMérieux, Marcy-l’Étoile, France). We further used *β-tubulin* gene sequencing to confirm the species identification. *A. tubingensis* (n = 27, 50.0%) and *A. niger* (n = 25, 46.3%) were the most prevalent species among clinical isolates. Two isolates of *A. welwitschiae* were identified.

### 3.2. In Vitro Activity of Azoles Against Section Nigri

**Figure 1:**
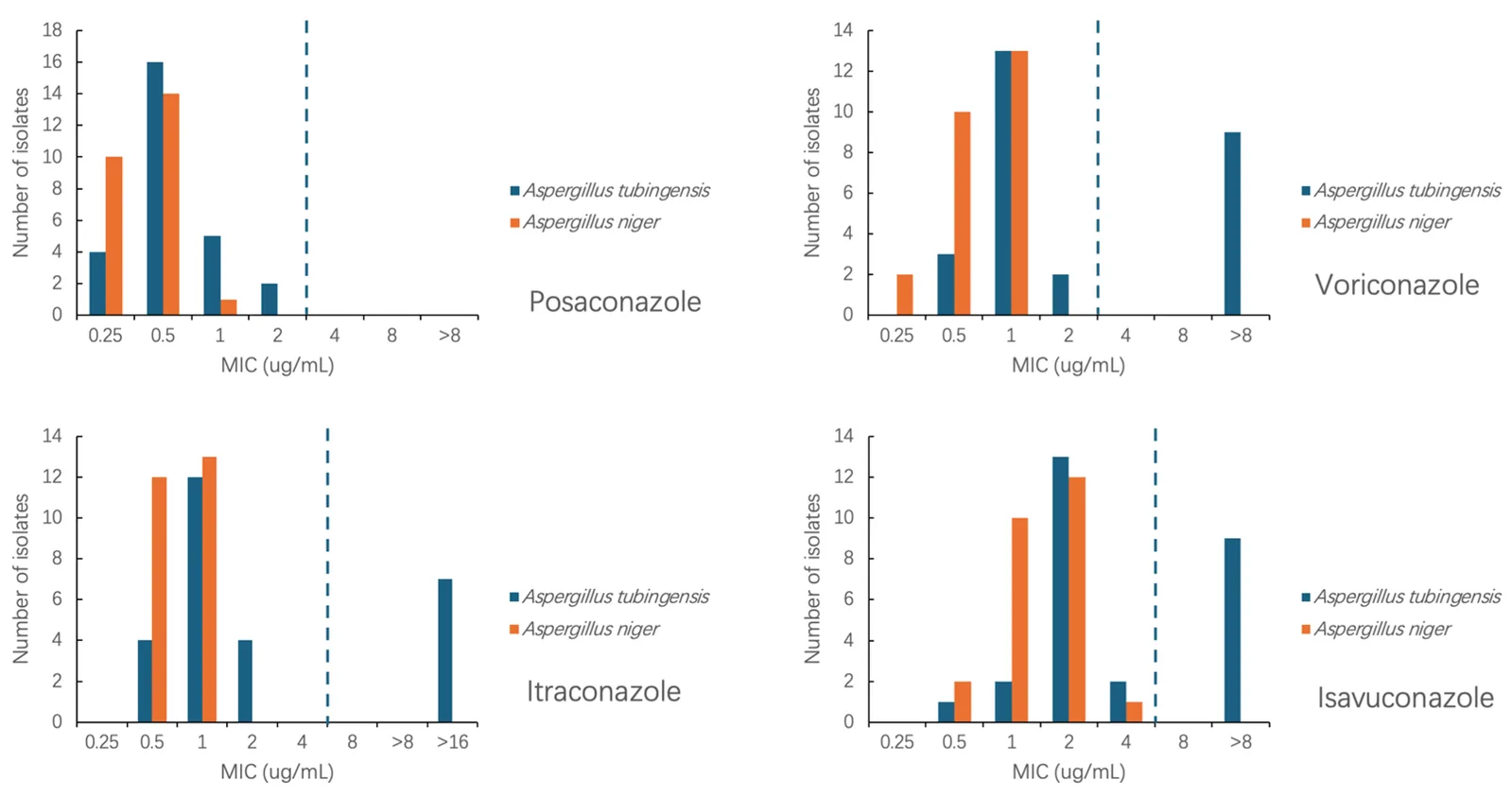
Distribution of posaconazole, voriconazole, itraconazole, and isavuconazole MICs for *A. tubingensis* and *A. niger*. The dotted lines indicate the CLSI ECVs for *A. niger*.

We determined MICs for itraconazole, voriconazole, posaconazole, and isavuconazole in all 54 *Aspergillus* section *Nigri* isolates. All *A. niger* and *A. welwitschiae* isolates were susceptible to these azoles. MIC ranges were 0.25 to 1 μg/mL for posaconazole and voriconazole, 0.5 to 1 μg/mL for itraconazole, and 0.5–4 μg/mL for isavuconazole. Among the 27 *A. tubingensis* isolates, nine (33.3%) had MICs above the ECVs for at least one azole. Elevated MICs were most frequent for voriconazole (n = 9) and isavuconazole (n = 9). Seven *A. tubingensis* isolates had elevated MICs to voriconazole, isavuconazole, and itraconazole; two showed elevated MICs to both voriconazole and isavuconazole (Figure 1).

### 3.3. Comparison of Published Studies on Azole-Resistance Rates in Aspergillus Section Nigri Isolates

We compared the present study’s results to others; the current posaconazole-resistance rate in *A. tubingensis* corresponded well with studies from Japan, Iran, France, and Italy. However, published azole-resistance rates (voriconazole, itraconazole, and isavuconazole) in *A. tubingensis* have varied internationally (Table 1). In contrast, *A. niger* and *A. welwitschiae* displayed much lower MIC values among isolates in the present study and published studies.

### 3.4. Analysis of A. tubingensis cyp51A Variant

Across all resistant isolates, we observed three *cyp51A* amino acid substitutions compared with azole-susceptible *A. tubingensis* strain P53-27176 (Table 2). Azole-resistant *A. tubingensis* strain P53-27434 revealed an alteration at codon 119 (Y119F), which exhibited elevated MICs to both voriconazole and isavuconazole. P9-27838 also had a mutation at codon 119 (Y119H). A nonsynonymous mutation T321A was found in P36-38063. These two isolates had elevated MICs for voriconazole, isavuconazole, and itraconazole. The remaining six resistant isolates had a wild-type cyp51A sequence.

## 4. Discussion

This study provided us with a regional report on the species distribution and antifungal resistance of section *Nigri* species in Eastern China. Different species exhibited remarkable differences in susceptibility to medical triazoles. In light of these situations, accurate species identification and antifungal susceptibility testing were crucial for effective disease management.

Half of the *Aspergillus* section *Nigri* recovered from respiratory specimens in Zhongshan Hospital, Fudan University in Shanghai, China, were identified as *A. tubingensis.* This finding agrees with reports revealing *A. tubingensis* as the most frequently isolated section *Nigri* species recovered from pulmonary specimens in France and Southern California [20,24]. Furthermore, this study was conducted during the COVID-19 pandemic, and nearly 20% of patients enrolled were diagnosed with COVID-19-associated pulmonary aspergillosis or influenza-associated pulmonary aspergillosis. The majority of these patients were admitted to the intensive care unit (ICU), with an overall mortality rate of approximately 60%. Consistent with our findings, case reports from Iran and Saudi Arabia documented COVID-19 with *A. tubingensis* coinfection [25,26]. Critically ill COVID-19 patients are at high risk for developing invasive fungal coinfections. These findings highlight *A. tubingensis* as an underrecognized pathogen of pulmonary aspergillosis. Further research is warranted from a public health perspective and to inform management strategies.

Furthermore, one-third of *A. tubingensis* isolates in our study had MICs above the ECVs for at least one medical triazole. Li et al. conducted a Chinese analysis of the *Aspergillus* section *Nigri* isolated from environmental and clinical samples between 1997 and 2014. All of these isolates had low MICs for triazoles [19]. The elevated MICs in *A. tubingensis* might be related to increased exposure to agricultural triazole fungicides [24]. Such environmental selection has contributed to azole resistance in *A. fumigatus* and has become a growing global public health concern [27]. In our study, itraconazole, voriconazole, and isavuconazole resistance was found in 26%, 33.3%, and 33.3% of A. tubingensis isolates, respectively. All of our isolates were sensitive to posaconazole. Previous studies reported different azole-resistance rates. Howard et al. reported that 90% of *A. tubingensis* isolates were resistant to itraconazole. Among those itraconazole-resistant section *Nigri* isolates, only 6% and 12% were resistant to posaconazole and voriconazole, respectively [28]. A study in Japan showed that 46.2% to 89.7% of *A. tubingensis* isolates were less susceptible to itraconazole and/or voriconazole [11]. Carrara et al. conducted a multi-centered surveillance study in France. This study reported that the majority of *A. tubingensis* isolates were susceptible to posaconazole (96.1%) and voriconazole (90.2%), whereas only 45.1% of isolates were susceptible to itraconazole [23]. In contrast, a recent study conducted in Southern California showed reduced susceptibility to posaconazole and itraconazole in *A. tubingensis* [24]. Hence, the frequency of azole resistance in *A. tubingensis* varied across geographic regions (Table 1). This reinforces the need to perform routine susceptibility testing for clinical *Aspergillus* section *Nigri*, especially its related cryptic species. Regional surveillance studies are crucial to address the critical gap and guide effective treatment.

The underlying drug resistance mechanism in the section *Nigri* has not yet been fully explored. We identified several *cyp51A* amino acid substitutions, namely Y119F, Y119H, and T321A. The T321A variant has been observed in *A. tubingensis* isolates exhibiting cross-resistance to voriconazole, itraconazole, and isavuconazole. This mutation has been previously reported in azole-resistant isolates [11,24]. However, it was classified as a nonsynonymous mutation since it was also present in azole-susceptible isolates [13]. Furthermore, two novel amino acid alterations, Y119H and Y119F, were detected in azole-resistant *A. tubingensis* isolates. The tyrosine residue at position 119 (Y119) of CYP51A is highly conserved among most fungi, including *Aspergillus* species, which suggests a critical functional and structural role in enzymatic activity [29]. Y119F substitution in the *cyp51A* gene has been previously reported as a key player in azole resistance in *A. flavus* [30]. Zhou et al. observed that the Y119F mutation reduced the conformational stability of the CYP51A protein, which in turn impairs azole binding. *A. flavus* isolate carrying the Y119F substitution exhibited reduced susceptibility to isavuconazole and voriconazole, while posaconazole and itraconazole remained sensitive [29]. This susceptibility pattern was consistent with our findings (P53-27434), suggesting Y119F may also represent a primary resistance-conferring mutation in *A. tubingensis*. CYP51A protein sequences show high similarity in *Aspergillus* species, and Y119 is highly conserved. Based on observations of previous studies on the Y121F mutation in *A. fumigatus* (corresponding to the Y119F mutation found in *A. tubingensis* and *A. flavus*) and the Y119F mutation in *A. flavus*, we speculate that the Y119F substitution in *A. tubingensis* may reduce the flexibility of specific regions around the heme-accessible channel and residue 119 [29,31,32]. The mutant CYP51A protein could exhibit a reduced average number of hydrogen bonds, potentially destabilizing the active center of the protein. Moreover, it is plausible that the Y119F mutation could lead to shortening and narrowing of the main access tunnels to the heme active site. These structural alterations might hinder substrate access or binding and thereby decrease azole susceptibility. In contrast, Y119H substitution replaces tyrosine with histidine. The imidazole ring might reorganize and destabilize the active-site microenvironment by rewiring residual hydrogen bond connections and altering electrostatic distribution within the heme pocket. Furthermore, we speculated that this variant could indirectly disrupt the coordination stability between triazole nitrogen atoms and heme iron. The flexible orientation of the imidazole side chain could introduce subtle steric perturbations and local residue reorientation within the ligand-access tunnel. We speculate that this substitution could act as an accessory mutation rather than an independent driver of azole resistance in *A. tubingensis.* These structural hypotheses remain unvalidated in the present study. In silico protein structure analysis, molecular dynamics simulation, tunnel analysis, and experimental structural determination will be essential to confirm our hypothesis. Furthermore, we characterized *A. tubingensis* strains P53-27434 and P53-27176 isolated from a patient with chronic pulmonary aspergillosis. Initially, azole-susceptible strain P53-27176 was isolated from this patient after receiving voriconazole treatment for one month. Over a period of two months, P53-27434 harboring the Y119F mutation was isolated from this patient. We hypothesized that the Y119F substitution might be associated with antifungal selection pressure. In-depth investigations, including antibiotic stress testing, will be conducted to better understand the physiological and metabolic responses when the azole-susceptible strain P53-27176 is exposed to sublethal levels of voriconazole. Further whole-genome sequencing of these two strains, CYP51A protein analysis, and tunnel analysis are required for elucidation.

This study also has some limitations. Six of the nine azole-resistant *A. tubingensis* isolates possessed a wild-type *cyp51A* sequence, which reinforced the multifactorial nature of azole resistance in *A. tubingensis*. A study conducted by Sen et al. identified overexpression of efflux pump genes (*mdr1* and *mfs* genes) as a potential driver of azole resistance [13]. A further whole-genome sequencing-based approach is warranted to reveal the complex azole resistance mechanisms in *A. tubingensis*. Our study was also limited by its single-center nature. The present study could provide a snapshot of the clinical *Aspergillus* section *Nigri* species profile from Eastern China. However, the generalizability of our findings to other regions of China or the world remains uncertain. We were also constrained by the absence of species-specific CBPs and ECVs for cryptic species including *A. tubingensis*. We could not determine whether the elevated MICs observed in *A. tubingensis* isolates fell outside the species’ wild-type distribution. Consequently, this introduced a layer of uncertainty regarding the “susceptible/wild-type” versus “resistant/non-wild-type” classification for these species. Further multicenter surveillance programs are essential to establish species-specific CBPs/ECVs for these emerging pathogens. Finally, the present study was primarily a microbiological and molecular investigation focused on clinical section *Nigri* isolates. Clinical outcome data in our cohort were limited to basic survival status. Larger prospective studies with comprehensive clinical metadata are needed to explore the correlation between the identified mutations and patient clinical outcomes.

## 5. Conclusions

In conclusion, *A. tubingensis* constituted a significant proportion of *Aspergillus* section *Nigri* recovered from respiratory specimens in Eastern China. Variable susceptibility patterns between species highlight the importance of accurate species identification. Our findings also underscore the importance of antifungal susceptibility testing. In order to reveal the mechanism of azole resistance, we identified the novel Y119F mutation in the cyp51A gene of A. tubingensis. Although the correlation between this substitution and azole resistance is not fully proven, indirect evidence is available.

## Figures and Tables

**Table 1:** Comparison of published studies on azole-resistance rates in *Aspergillus* section *Nigri* isolates.

Organism	Author (Year) [Reference]	Location	Number of Isolates	Method	MIC Range (% WT)			
					POS	VOR	IZ	ISA
***Aspergillus tubingensis***	This study	China	27	YeastOne	0.25–2 (100%)	0.5–>8 (66.7%)	0.5–>16 (74%)	0.5–>8 (66.7%)
	Li et al. (2015) [19]	China	23	CLSI	0.125–1 (100%)	0.25–2 (100%)	1 (100%)	NA
	Gautier et al. (2016) [20]	France	36	Etest	0.047–0.75 (100%)	0.125–1 (100%)	0.38–32 (NA)	NA
	Iatta et al. (2016) [21]	Italy	32	CLSI	0.25–1 (100%)	0.5–2 (100%)	0.5–2 (100%)	NA
	Badali et al. (2016) [22]	Iran	49	CLSI	0.016–0.125 (100%)	0.063–2 (100%)	0.125–>16 (77.6%)	NA
	Hashimoto et al. (2017) [11]	Japan	39	CLSI	NA	2–>8 (10.3%)	1–>8 (53.8%)	NA
	Carrara et al. (2020) [23]	France	51	EUCAST	0.12–4 (96.1%)	0.5–4 (90.2%)	0.5–16 (45.1%)	1–16 (60.8%)
			43	Etest	0.006–0.25 (100%)	0.008–0.25 (100%)	0.004–4 (100%)	0.03–1 (100%)
	Pfaller et al. (2024) [12]	Global	5	CLSI	0.5–2 (100%)	1–4 (80%)	2–>8 (80%)	2–8 (80%)
	Wang et al. (2025) [24]	USA	59	Etest	0.19–1.5 (45.8%)	0.047–3 (81.4%)	0.5–32 (57.6%)	0.25–8 (93.2%)
***Aspergillus niger***	This study	China	25	YeastOne	0.25–1 (100%)	0.25–1 (100%)	0.5–1 (100%)	0.5–4 (100%)
	Li et al. (2015) [19]	China	20	CLSI	0.125–0.25 (100%)	0.125–0.5 (100%)	0.5–1 (100%)	NA
	Gautier et al. (2016) [20]	France	36	Etest	0.047–1 (100%)	0.064–1 (100%)	0.25–24 (NA)	NA
	Iatta et al. (2016) [21]	Italy	3	CLSI	0.25–1 (100%)	0.25–2 (100%)	0.25–2 (100%)	NA
	Badali et al. (2016) [22]	Iran	72	CLSI	0.016–0.25 (100%)	0.125–>16 (76.4%)	0.125–>16 (79.2%)	NA
	Hashimoto et al. (2017) [11]	Japan	20	CLSI	NA	0.5–8 (45%)	0.25–8 (90%)	NA
	Carrara et al. (2020) [23]	France	7	EUCAST	0.25–1 (100%)	0.25–2 (100%)	0.5–2 (100%)	2–8 (28.6%)
			5	Etest	0.03–0.25 (100%)	0.03–0.25 (100%)	0.5–1.5 (100%)	NA
	Pfaller et al. (2024) [12]	Global	77	CLSI	0.25–1 (100%)	0.25–4 (98.7%)	0.25–8 (97.4%)	0.5–>8 (96.1%)
***Aspergillus welwitschiae***	This study	China	2	YeastOne	0.5 (100%)	1 (100%)	1 (100%)	2–4 (100%)
	Hashimoto et al. (2017) [11]	Japan	59	CLSI	NA	0.5–4 (81.4%)	0.25–8 (98.3%)	NA
	Carrara et al. (2020) [23]	France	50	EUCAST	0.12–1 (100%)	0.5–2 (100%)	0.25–16 (84%)	0.5–8 (88%)
			40	Etest	0.008–0.5 (100%)	0.01–0.25 (100%)	0.12–2 (100%)	0.09–1 (100%)

**Table 2:** Mycological characteristics of azole-resistant *A. tubingensis* strains and clinical profiles of patients harboring resistant strains.

Case	Age/ Gender	Sample Type	Clinical Diagnosis	Outcome	Strain	MIC (ug/mL)				*cyp51A* Mutation
						POS	VOR	IZ	ISA	
**1**	76/male	Sputum	Chronic pulmonary aspergillosis	Discharged	P9-27838	1	**>8**	**>16**	**>8**	Y119H
**2**	76/male	Sputum	COVID-19- associated pulmonary aspergillosis	Discharged	P12-27321	1	**>8**	**>16**	**>8**	-
**3**	59/male	Sputum	Lung cancer and liver cancer	Died	P24-20677	1	**>8**	**>16**	**>8**	-
**4**	65/male	Sputum	Chronic obstructive pulmonary disease	Discharged	P25-27557	2	**>8**	**>16**	**>8**	-
**5**	73/female	Sputum	Chronic pulmonary aspergillosis	Discharged	P27-27803	0.5	**>8**	2	**>8**	-
**6**	49/male	Sputum	Ventilator- associated pneumonia and probable invasive aspergillosis	Discharged	P32-27083	1	**>8**	**>16**	**>8**	-
**7**	60/male	Sputum	Stomach cancer	Discharged	P9-28063	2	**>8**	**>16**	**>8**	T321A
**8**	74/female	Sputum	Sjogren’s syndrome	Discharged	P51-222798	1	**>8**	**>16**	**>8**	-
**9**	67/male	Sputum	Chronic pulmonary aspergillosis	Discharged	P53-27176	0.5	1	1	2	-
		Sputum			P53-27434	0.5	**>8**	1	**>8**	Y119F

## Data Availability

The raw data supporting the conclusions of this study will be made available by the corresponding author upon reasonable request.

## Abbreviations

The following abbreviations are used in this manuscript:

MIC	Minimum Inhibitory Concentration
IA	Invasive Aspergillosis
CPA	Chronic Pulmonary Aspergillosis
ECV	Epidemiological Cutoff Values
CLSI	Clinical and Laboratory Standards Institute
